# Generation of Novel *Traj18*-Deficient Mice Lacking Vα14 Natural Killer T Cells with an Undisturbed T Cell Receptor α-Chain Repertoire

**DOI:** 10.1371/journal.pone.0153347

**Published:** 2016-04-11

**Authors:** Nyambayar Dashtsoodol, Tomokuni Shigeura, Ritsuko Ozawa, Michishige Harada, Satoshi Kojo, Takashi Watanabe, Haruhiko Koseki, Manabu Nakayama, Osamu Ohara, Masaru Taniguchi

**Affiliations:** 1 Laboratory for Immune Regulation, RIKEN Center for Integrative Medical Sciences (IMS), Yokohama, Kanagawa, 230–0045, Japan; 2 Laboratory for Integrative Genomics, RIKEN Center for Integrative Medical Sciences (IMS), Yokohama, Kanagawa, 230–0045, Japan; 3 Laboratory for Developmental Genetics, RIKEN Center for Integrative Medical Sciences (IMS), Yokohama, Kanagawa, 230–0045, Japan; 4 Department of Human Genome Research, Kazusa DNA Research Institute, Kisarazu, Chiba, 292–0818, Japan; 5 Core Research Laboratory, Mongolian National University of Medical Sciences (MNUMS), Ulaanbaatar, 14210, Mongolia; Oklahoma Medical Research Foundation, UNITED STATES

## Abstract

Invariant Vα14 natural killer T (NKT) cells, characterized by the expression of a single invariant T cell receptor (TCR) α chain encoded by rearranged *Trav11* (Vα14)*-Traj18* (Jα18) gene segments in mice, and *TRAV10* (Vα24)*-TRAJ18* (Jα18) in humans, mediate adjuvant effects to activate various effector cell types in both innate and adaptive immune systems that facilitates the potent antitumor effects. It was recently reported that the Jα18-deficient mouse described by our group in 1997 harbors perturbed TCRα repertoire, which raised concerns regarding the validity of some of the experimental conclusions that have been made using this mouse line. To resolve this concern, we generated a novel *Traj18*-deficient mouse line by specifically targeting the *Traj18* gene segment using Cre-Lox approach. Here we showed the newly generated *Traj18*-deficient mouse has, apart from the absence of *Traj18*, an undisturbed TCRα chain repertoire by using next generation sequencing and by detecting normal generation of Vα19Jα33 expressing mucosal associated invariant T cells, whose development was abrogated in the originally described Jα18-KO mice. We also demonstrated here the definitive requirement for NKT cells in the protection against tumors and their potent adjuvant effects on antigen-specific CD8 T cells.

## Introduction

Invariant Vα14 natural killer T (NKT) cells are a unique lymphocyte subset characterized by the expression of a single invariant T cell receptor (TCR) α chain encoded by rearranged *Trav11* (Vα14)*-Traj18* (Jα18) gene segments in mice, and *TRAV10* (Vα24)*-TRAJ18* (Jα18) in humans. Both human and mouse NKT cells recognize glycolipid ligands, such as α-galactosylceramide (αGalCer), presented by the monomorphic major histocompatibility complex (MHC)-like CD1d molecule. NKT cells mediate many important immune regulatory functions, such as protection against pathogens and tumors, maintenance of transplantation tolerance, prevention of autoimmune disease development, and regulation of allergic responses. One of most well-studied and important features of NKT cells is their adjuvant activity, which can induce activation of both adaptive and innate arms of the immune response. For example, in the cancer setting NKT-activated adaptive CD8 T and innate NK effector cells can kill MHC-positive and MHC-negative tumor cells, respectively, thus effectively eliminating the tumor [1,2].

Many investigators have used the originally established Jα18-deficient mice, described by our group back in 1997, which paved the way to understanding the functional roles of NKT cells in various experimental settings [3]. However, a recently published correspondence described a perturbed TCRα repertoire in these mice due to suppressed transcription of *Traj* gene segments upstream of *Traj18* [4]. This has raised concerns regarding the validity of some of the experimental conclusions that have been made using this mouse line.

In this study, we established a novel *Traj18*-deficient mouse line lacking specifically invariant NKT cell lineage that could be used as an undisputed mouse model for future investigations in the NKT cell field, and also validated cardinal features of NKT cells related with potent tumor rejection and adjuvant effects on antigen-specific adaptive immunity.

## Materials and Methods

### Generation of *Traj18*-deficient mice

**Fig 1:**
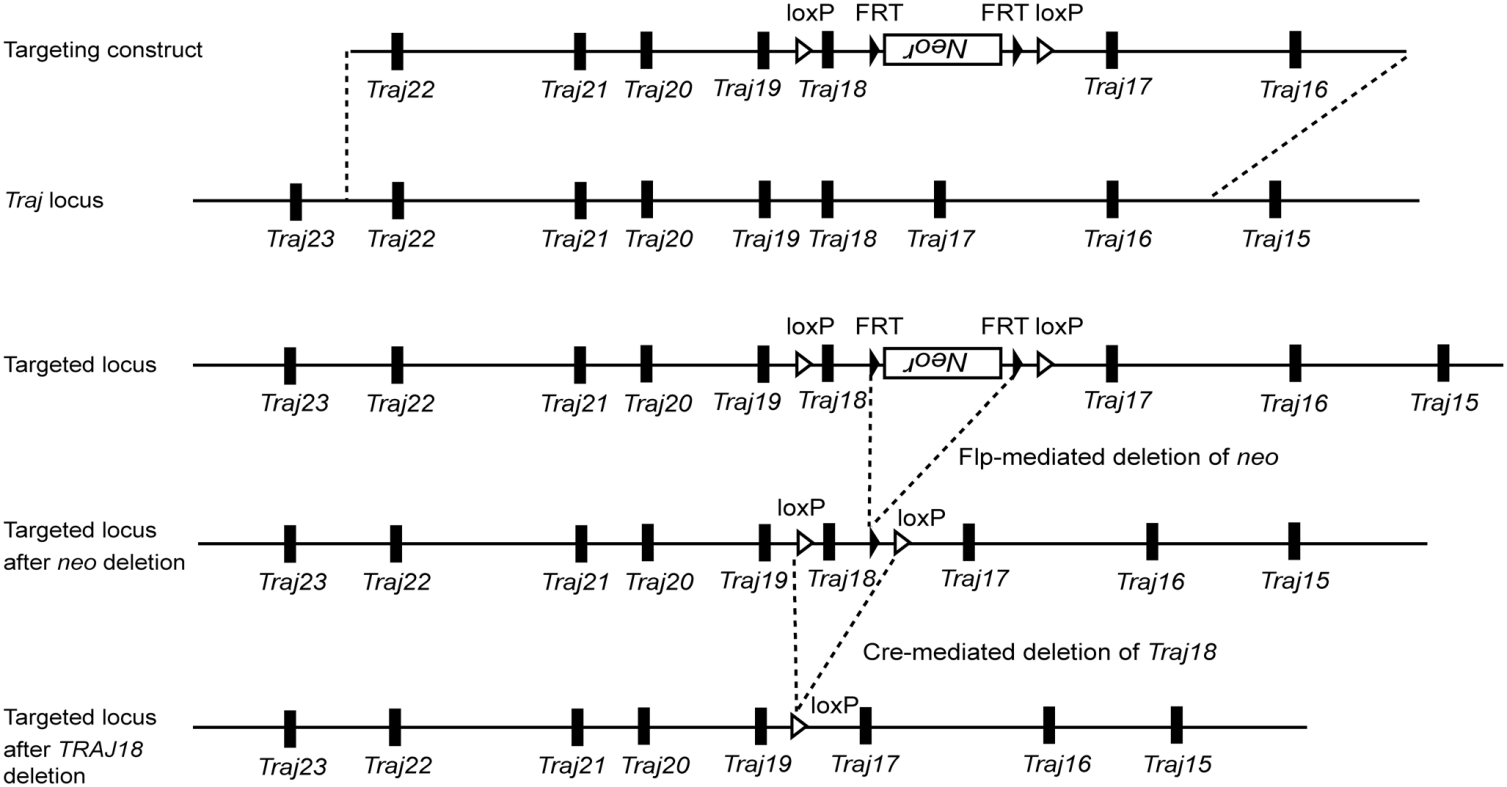
Generation of novel *Traj18*-deficient mice. Schematic representation of a *Traj18* region targeting construct, *Traj18* region before and after homologous recombination, and the genomic locus after FLP- and Cre-mediated deletions of the neomycin resistance gene and *Traj18*, respectively.

A *Traj18* region targeting vector was constructed as shown in Fig 1, and then transduced into B6JN/1 mouse embryonic stem cells, derived from a (C57BL6/J Jcl) x (C57BL6/N Jcl) embryo, that were established at the RIKEN IMS animal facility, and were further selected using conventional methods. The FRT-flanked neomycin cassette was removed by mating of chimeric mice to CAG-FLP recombinase transgenic mice, and the loxP-flanked *Traj18* region was subsequently deleted by breeding to CAG-Cre recombinase transgenic mice. The resultant mice were mated to derive homozygous *Traj18*-deficient mice.

### Mice

Wild-type (WT) C57BL/6 (B6) mice were purchased from Charles River Laboratories, *Jα18*^-/-^ and *Cd1d1*^-/-^*Cd1d2*^-/-^ mouse lines were described [3,5]. Mice were maintained in the animal facility of RIKEN IMS under specific pathogen-free conditions and were used at 8–10 weeks of age. All animal experiments were approved by RIKEN Animal Care and Use Committee.

### Cell preparation and flow cytometry

FITC, PE, PerCP-Cy5.5, PE-Cy7, APC, or BV421 conjugated mAbs specific for B220 (RA3-6B2), CD3ε (145-2C11), CD4 (GK1.5), CD8α (53–6.7), TCRβ (H57-597) were purchased from BD Biosciences or BioLegend or eBioscience. Thymocytes, splenocytes and liver mononuclear cells were prepared and stained with αGalCer/CD1d dimers as described [6,7]. Lungs were prepared as described [8]. Cell staining was performed after blocking with anti-FcR (2.4G2). Forward light-scatter gating and 7-AAD staining were used to gate out dead cells. Samples were analyzed using FACSCanto or FACSAria instruments (BD Biosciences), and data were analyzed with FlowJo (Tree Star).

### Systemic activation of NKT cells with αGalCer

Two micrograms of αGalCer were injected into the tail veins of WT or *Traj18*^*-/-*^ mice or *Cd1d1*^-/-^*Cd1d2*^-/-^ mice. Blood serum was collected at 3 or 24 h post-injection and cytokine levels were measured using a cytokine bead assay according to the manufacturer's instructions (BD Biosciences). Data were acquired using FACS Canto flow cytometer (BD Biosciences), and analyzed with FCAP Array^™^ software (Soft Flow).

### TCR sequencing

CD4^+^CD8^+^ thymocytes from WT or *Traj18*-deficient mice were sorted on a FACS Aria cell sorter (BD Biosciences) with post-sort purity above 99%. Cells were lysed and RNA was extracted with RNeasy kit (Qiagen). cDNA was prepared with Superscript First-Strand SuperMix (Life Technologies). The following primers with Illumina adaptor sequences at the 5' ends were used to amplify the *Trav11-Trac* transcripts (*Trav11* sense, 5'-GTCCTCAGTCCCTGGTTGTC-3' and *Trac* anti-sense, 5'-AGGGTGCTGTCCTGAGACCGA-3') using a KAPA HiFi high fidelity PCR mix (Kapa Biosystems). PCR products were purified and sequenced on a MiSeq system with a MiSeq Reagent Kit v3, 600 cycles (Illumina). Mouse TCR Jα regions were analyzed using IMGT/HighV-QUEST from the IMGT (international ImMunoGeneTics information system) database [9].

### B16 melanoma metastasis model

Mice were anesthetized and the spleen was surgically removed on day 0 after intrasplenic inoculation of B16 melanoma (5×10^5^) cells. Two days after inoculation of B16, mice were injected intravenously with αGalCer-pulsed bone marrow derived DCs (5×10^5^). The mice were sacrificed on day 14 after B16 inoculation, and the liver was visually evaluated for B16 metastases.

### αGalCer-induced adjuvant activity on OVA-specific CD8 T cells

To identify OVA-specific T cells expanded upon NKT stimulation *in vivo*, splenocytes were prepared according to a published report [10] with some minor modifications. In brief, splenocytes pulsed with OVA peptide (Worthington Biochemical) were administered intravenously with 2 μg of αGalCer into the tail veins of recipient mice. Seven days later the mice were sacrificed and splenocytes were directly assessed for the presence of OVA-specific CD8 T cells using anti-CD3, CD8 (BD Biosciences) and T-Select H-2Kb OVA Tetramer-SIINFEKL-APC (MBL), or were primed *in vitro* with or without 1 μM OVA_257–264_ peptide (SIINFEKL) (Abbiotec) for 6 h in the presence of GolgiPlug (BD Biosciences). The cells were then stained with cell-surface markers, fixed with Cytofix/Cytoperm Plus permeabilization kit (BD Biosciences), and stained with an anti-IFN-γ mAb (BD Biosciences).

### Real-time quantitative RT-PCR

Total RNA was prepared from sorted lung αGC/CD1d^-^ TCRβ^+^ T lymphocytes with RNeasy Plus Micro kit (Qiagen) and was reverse transcribed using Superscript VILO master mix (Life Technologies). The real-time quantitative RT-PCR was performed on LightCycler 480 instrument (Roche) with the Universal ProbeLibrary (UPL) probe #13 (Roche) and following primer pairs: *Trav1* sense, 5'-CTTTCCTGAGCCGCTCGAA-3' and *Traj33* anti-sense, 5'-CTTGGTCCCAGAGCCCC-3'. The relative gene expression was calculated using 2^-ΔΔCt^ method, where the expression level of *Trac*, detected with the UPL probe #18 together with *Trac* sense, 5'-ATGCCACGTTGACTGAGAAA-3'; *Trac* anti-sense, 5'-AGCAGGTTAAATCCGGCTACT-3', served as an internal control.

### Statistical analysis

Statistical analyses were performed using Prism 6.0 software (GraphPad). Two-tailed unpaired *t* test was used to compare two groups. *P*-values less than 0.05 were considered statistically significant.

## Results and Discussion

### Generation of novel *Traj18*-deficient mice lacking Vα14 NKT cells

In the original Jα18-deficient mouse line the *PGK-neo*^*r*^ selection cassette from the targeting vector was retained in the genome, prompting Bedel, et al. to speculate that the *neo* transcription was causing the abnormal usage of *Traj* gene segments [3,4]. To circumvent this problem, in the new mouse strain we used Cre- and Flp-mediated site-specific recombinase technologies to specifically and cleanly delete the *Traj18* gene segment (Fig 1).

**Fig 2:**
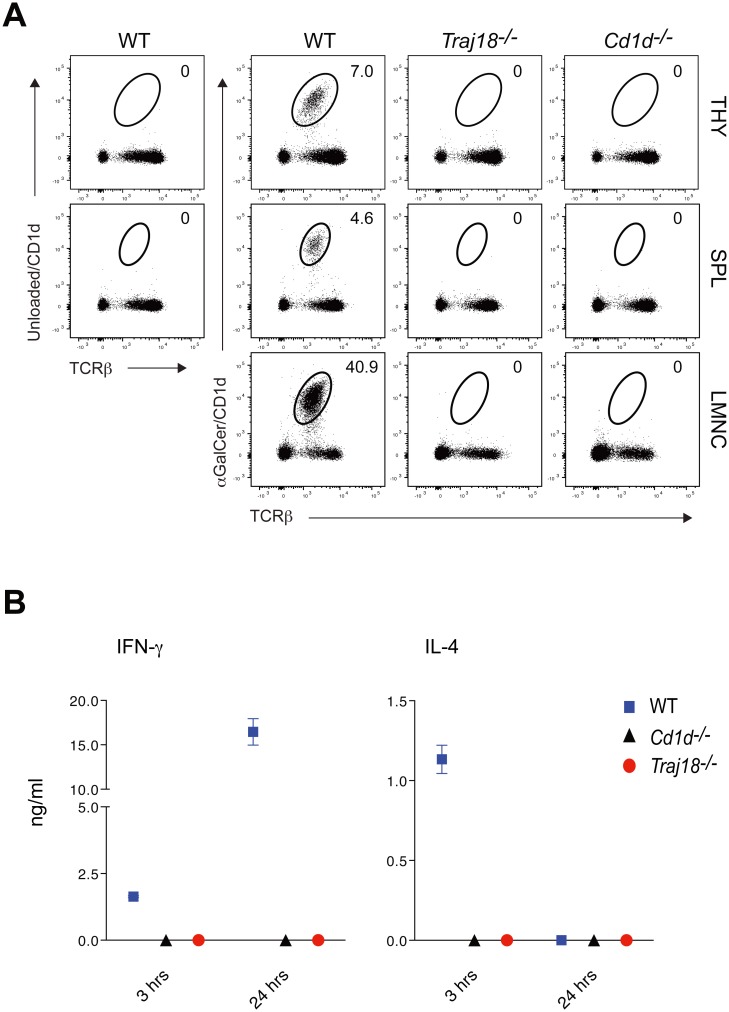
Newly generated *Traj18*-deficient mice lack Vα14 NKT cells. (A) Flow cytometry profiles of thymocytes, splenocytes and liver mononuclear cells from WT, *Traj18*^-/-^ and *Cd1d1*^-/-^*Cd1d2*^-/-^ mice. Unloaded CD1d dimer staining was used as a staining control. Numbers depict percentage of αGC/CD1d dimer^+^ TCRβ^+^ NKT cells among viable CD8^-^B220^-^ gated lymphocytes. The data are representative of three independent experiments. (B) *In vivo* cytokine production by NKT cells upon systemic activation with αGalCer administration. WT or *Cd1d1*^-/-^*Cd1d2*^-/-^ or *Traj18*^-/-^ mice were injected intravenously with 2 μg of αGalCer and blood plasma were collected after either 3 h and 24 h, and IFN-γ and IL-4 concentrations were measured using cytokine beads assay. Bars depict mean ± SEM of *n* = 3 mice per genotype analyzed. Data are representative of three experiments.

The resultant mouse line, termed *Traj18*^*-/-*^, was totally devoid of NKT cells in the thymus as well as in the spleen and liver, as revealed by staining with αGalCer-loaded CD1d dimer staining, where unloaded CD1d dimer staining served to exclude the background staining (Fig 2A).

Moreover, the intravenous injection of the NKT cell-specific agonist ligand αGalCer demonstrated the absence of functional NKT cells in *Traj18*^*-/-*^ mice, assessed by increased levels of IL-4 and IFN-γ, which were only detected in wild-type (WT) mice at 3 h and 24 h post-injection, respectively, but were undetectable in *Traj18*^*-/-*^ mice, or in *Cd1d1*^-/-^*Cd1d2*^-/-^ mice (Fig 2B). The latter mouse is another widely used model that has intact TCRα chain gene rearrangements but is deficient in both invariant and non-invariant NKT cells due to absence of the CD1d positive selector molecule [5].

### Undisturbed TCRα chain repertoire in *Traj18*-deficient mice

**Fig 3:**
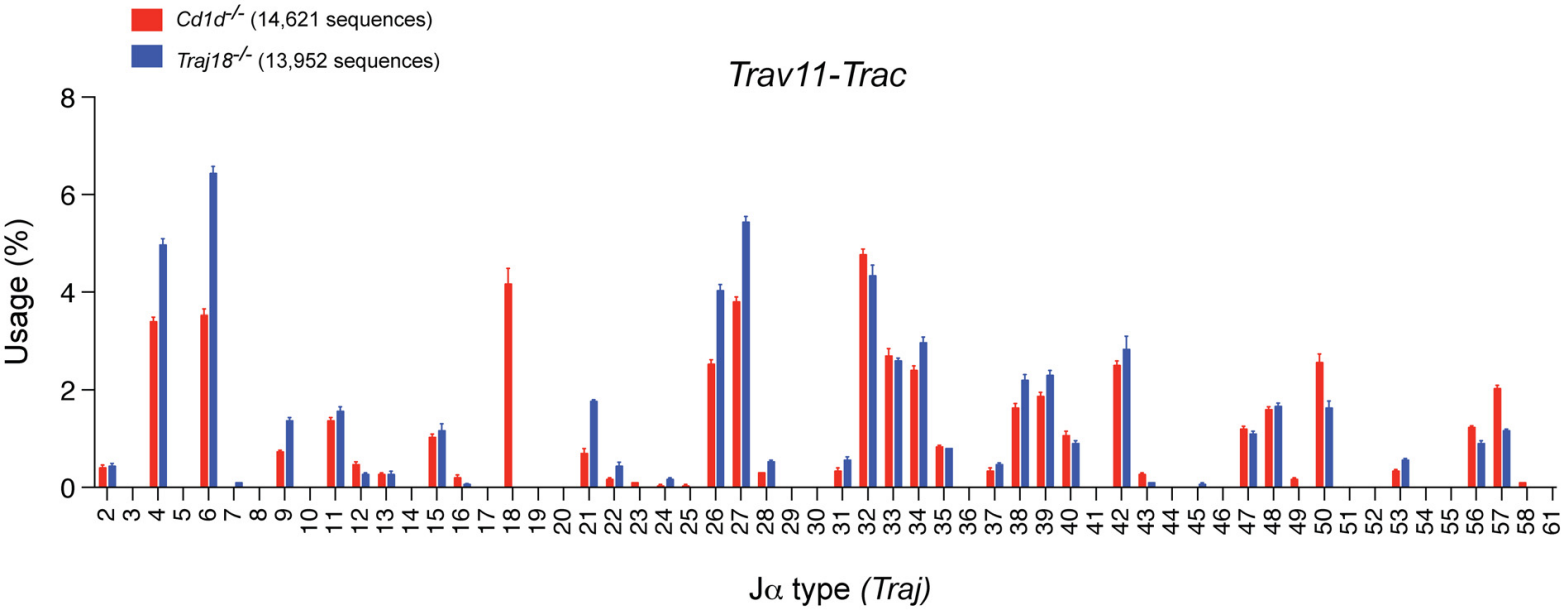
Undisturbed TCRα chain joining region usage in newly generated *Traj18*-deficient mice as revealed by next generation sequencing. Sequencing of TCRα chain joining region. PCR was carried out to amplify *Trav11-Trac* transcripts using cDNA prepared from sorted TCRβ^low^ CD4^+^CD8^+^ double-positive thymocytes from *Cd1d1*^-/-^*Cd1d2*^-/-^ (red bars) and *Traj18*^-/-^ (blue bars) mice. Bars depict mean ± SEM percentages of productive *Traj* gene segment rearrangements, and data are derived from three biologically independent samples per genotype. Numbers in parenthesis indicate the total number of sequences analyzed. The data are from one experiment.

Then we investigated the TCRα chain repertoire by sequencing *Trav11-Trac* transcripts prepared from sorted CD4^+^CD8^+^ double-positive (DP) thymocytes from newly established *Traj18*^*-/-*^ or from *Cd1d1*^-/-^*Cd1d2*^-/-^ mice. This analysis demonstrated that *Traj18*^*-/-*^ mice indeed specifically lack the *Traj18* gene segment while harboring an otherwise undisturbed TCRα chain repertoire that uses *Traj* gene segments upstream and downstream of *Traj18* similar to the situation in *Cd1d1*^-/-^*Cd1d2*^-/-^ mice (Fig 3).

### Normal development of MAIT cells with invariant Vα19Jα33 TCR α chain rearrangement in *Traj18*-deficient mice

**Fig 4:**
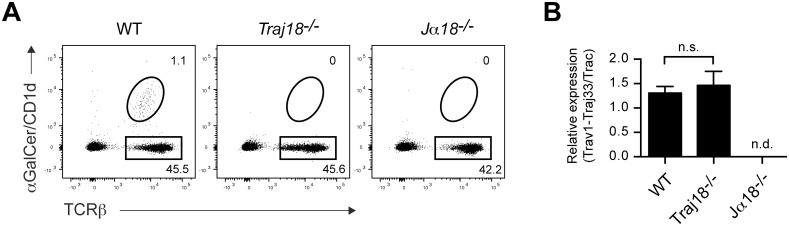
Normal development of MAIT cells with an invariant Vα19Jα33 in *Traj18*-deficient mice. (A) Sorting strategy of αGC/CD1d^-^ TCRβ^+^ lung T lymphocytes from WT, *Traj18*^*-/-*^ and previously generated *Jα18*^*-/-*^ mice. Numbers on FACS plots depict percentage of gated cells among viable 7-AAD^-^ B220^-^ lung lymphocytes. (B) Relative expression of Vα19Jα33 mRNA by real-time quantitative RT-PCR in sorted lung cells shown in (A). Gene expression was normalized using *Trac* as internal control. Bars depict mean ± SEM, n.s., not significant using unpaired *t* test. All data are representative of three independent experiments with a combined total of three mice per genotype.

To provide an additional proof of an undisturbed development of T cell lineages in *Traj18*^*-/-*^ mice, we assessed mucosal-associated invariant T (MAIT) cells representing a well-characterized MR1-restricted T cell lineage that uses invariant Vα19Jα33 in mice, and Vα7.2 joined to either Jα33 or Jα12 or Jα20 in humans [11]. It has been previously reported that the highest frequency of MAIT cells in B6 mice was detected in lung with the MR1-tetramer staining [12]. To this end, we sorted αGalCer/CD1d^-^ TCRβ^+^ lung T lymphocytes from WT or *Traj18*^*-/-*^ mice as well as from previously generated *Jα18*^*-/-*^ mice [3] that was reported to have a defective transcription of *Traj* gene segments upstream of *Traj18* [4] (Fig 4A), and assessed expressions of the invariant Vα19Jα33 TCRα chain encoded by *Trav1* and *Traj33* using real-time quantitative RT-PCR. Results clearly demonstrated the normal development of MAIT cells in *Traj18*^*-/-*^ mice as compared with control WT mice (Fig 4B), while *Jα18*^*-/-*^ mice lacked expression of *Trav1-Traj33*, indicating defective development of MAIT cells in the latter mouse line, which was in agreement with the previous report [4]. These results evidenced the normal development of T cells except for the absence of NKT cells in the newly generated *Traj18*^*-/-*^ mouse line.

### Failure to demonstrate NKT cell-mediated adjuvant activity on OVA-specific CD8 T cells in *Traj18*-deficient mice

**Fig 5:**
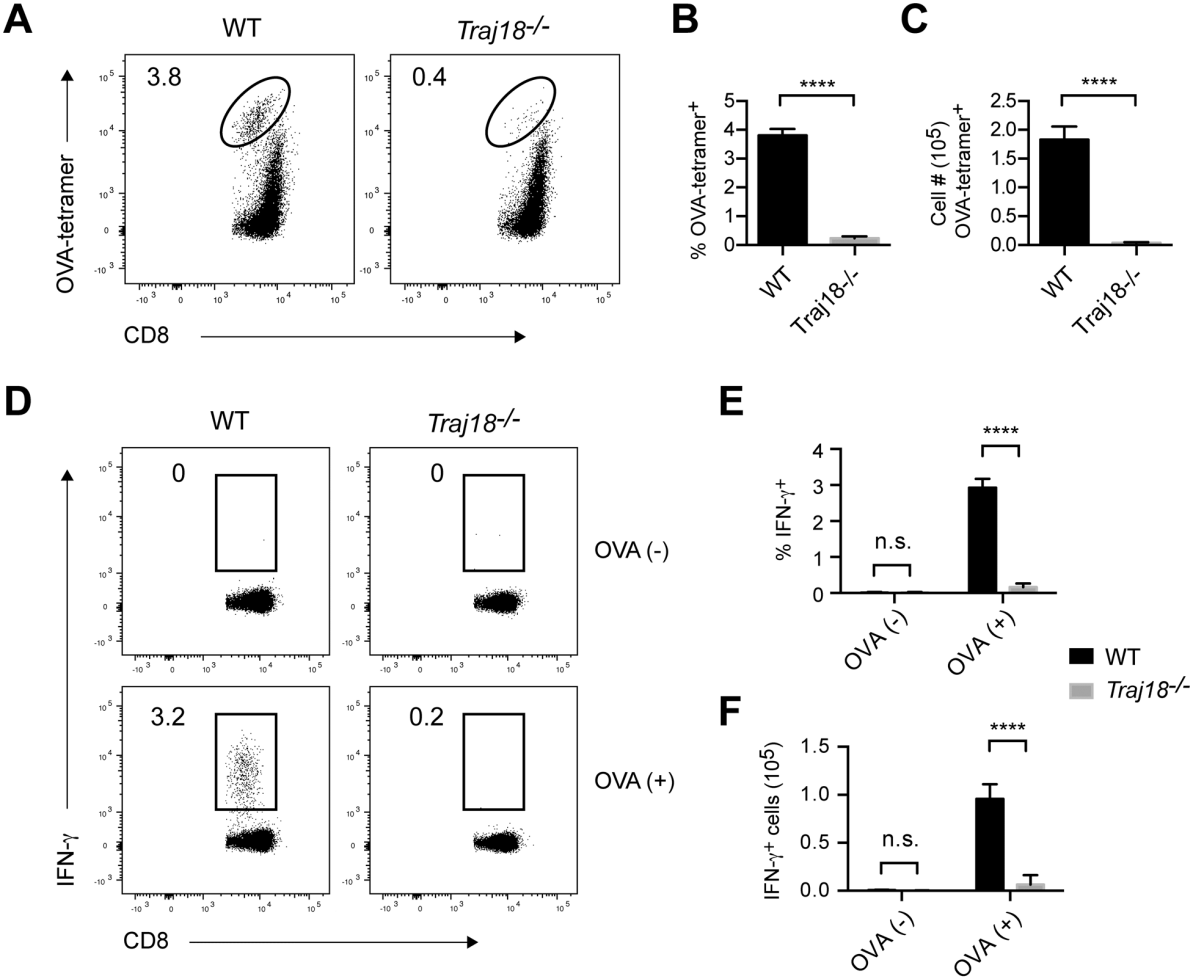
A validation of the adjuvant effect of Vα14 NKT cells using *Traj18*-deficient mice. (A) NKT cell-mediated adjuvant effect on the expansion of antigen-specific CD8 T cells. WT and *Traj18*^-/-^ mice were immunized with OVA antigen and αGalCer on day 0, and splenocytes were analyzed on day 7. Numbers on FACS plots represent percentage of OVA-tetramer positive cells among viable CD8 T cells. (B) Cell percentages and (C) numbers of OVA-tetramer positive cells gated as shown in A. Bars depict mean ± SEM for *n* = 9 mice per group. (D) NKT cell-mediated adjuvant effect on the activation of antigen-specific CD8 T cells. WT and *Traj18*^-/-^ mice were immunized with OVA antigen and αGalCer on day 0, and splenocytes were harvested on day 7. Cells then were cultured *in vitro* with or without OVA_257-264_ peptide for 6 h in the presence of GolgiPlug Protein Transport Inhibitor, and were stained with an IFN-γ mAb using Cytofix/Cytoperm kit. Numbers on FACS plots represent percentage of IFN-γ positive cells among CD8 T cells. (E) Percentages and (F) numbers of IFN-γ positive cells shown in D. Bars graphs depict mean ± SEM for *n* = 5 mice per group. All data shown are representative from three independent experiments. ****, *P* < 0.0001 using unpaired *t* test.

In order to confirm the adjuvant activity of NKT cells in the induction of antigen-specific CD8 T cells that presumably kill MHC-positive tumors, we assessed the frequency of such cytotoxic CD8 T cells upon administration of αGalCer into WT or *Traj18*^-/-^ mice. We observed significantly increased frequencies and numbers of OVA-specific CD8 T cells in WT but not in *Traj18*^-/-^ mice (Fig 5A, 5B and 5C) as well as clonotypic expansion and activation of IFN-γ producing antigen-specific CD8 T cells in WT but not in *Traj18*^-/-^ mice (Fig 5D, 5E and 5F). These data provide evidence proving the potent adjuvant effects of αGalCer activated NKT cells in the induction of antigen-specific CD8 T cells.

### Failure to demonstrate NKT cell-mediated anti-tumor effects in *Traj18*-deficient mice

Based on mouse and human studies, NKT cell-targeted adjuvant cell therapy was approved by the Japanese government for advanced non-small cell lung cancer in 2011, head and neck tumors in 2013, and post surgery stage IIA-IIIA non-small cell lung cancer in 2014 [13]. Therefore it was imperative to investigate the role of NKT cells in tumor rejection using the newly generated *Traj18*^*-/-*^ mice.

**Fig 6:**
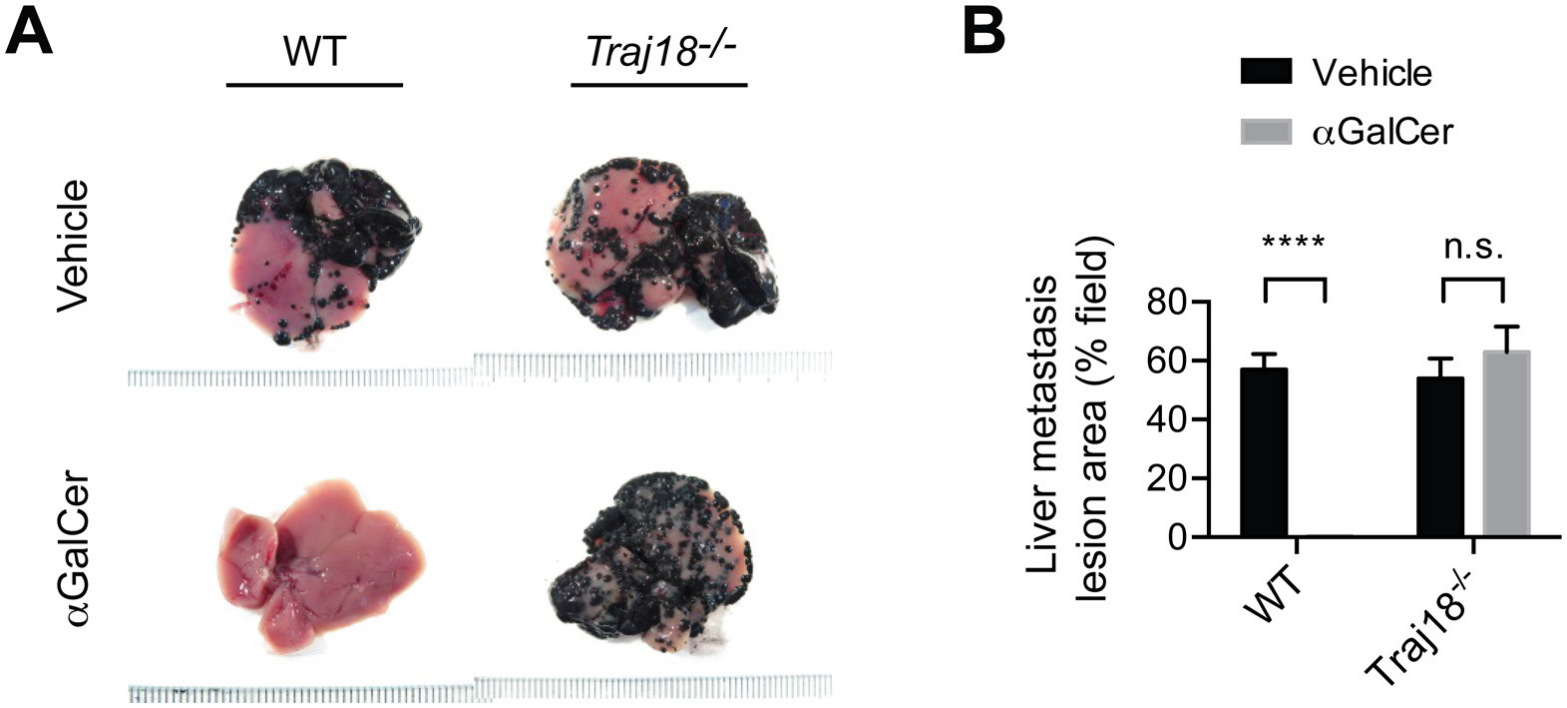
A validation of the requirement for Vα14 NKT cells for tumor rejection using *Traj18*-deficient mice. (A) Inhibition of B16 melanoma metastasis by specific activation of NKT cells with αGalCer. The anti-tumor effect of αGalCer-pulsed DC was assessed using the B16 melanoma liver metastasis model. Tumor cells were inoculated into WT or *Traj18*^-/-^ mice on day 0 and αGalCer-DC were injected intravenously on day 2. Representative images of liver tissues on day 14 are shown. (B) Liver metastasis area estimated by visual evaluation of percentage of the tumor field as shown in A. Bars depict mean ± SEM. Data are representative from three independent experiments with a combined total of 6–9 mice per group. ****, *P* < 0.0001 using unpaired *t* test.

To this end, we used a B16 melanoma liver metastasis model, where mice bearing metastatic melanoma nodules in the liver were treated by intravenous administration of αGalCer-pulsed dendritic cells (αGalCer-DC) as described previously [14]. This NKT cell-targeting immunotherapy resulted in the complete eradication of melanoma metastasis in WT but not in *Traj18*^*-/-*^ mice. Indeed the tumor growth in αGalCer-DC treated *Traj18*^*-/-*^ mice was similar to that in the vehicle-treated WT and *Traj18*^*-/-*^ mouse groups, demonstrating the absolute requirement for αGalCer-DC activated NKT cells in tumor rejection (Fig 6A and 6B).

Collectively, our present study clearly demonstrated the protective role of NKT cells against tumors by using the newly generated *Traj18*^-/-^ mice that specifically lack the NKT cell lineage and, apart from the absence of *Traj18*, have an undisturbed TCRα chain repertoire. Of note, while our manuscript was in preparation, another *Traj18*-deficient mouse line was reported, where the authors demonstrated a role of NKT cells in airway inflammation and resistance using OVA-induced and cockroach antigen-induced pulmonary inflammation models [15]. Thus, our results together with those from the Kronenberg group confirm the definitive requirement for NKT cells in both protection against tumors and regulation of allergic responses, using independently generated *Traj18*^-/-^ mouse strains with essentially normal TCR repertoires. The authors hope that both mouse lines will be useful for future investigations aimed in specifically assessing the role of NKT cells in various experimental settings.
